# Editorial: Affectivity Beyond the Skin

**DOI:** 10.3389/fpsyg.2018.01307

**Published:** 2018-07-25

**Authors:** Giovanna Colombetti, Joel Krueger, Tom Roberts

**Affiliations:** Department of Sociology, Philosophy, and Anthropology, University of Exeter, Exeter, United Kingdom

**Keywords:** affect, emotions, 4E cognition, embodied cognition, scaffolding, niche construction

A growing number of researchers in cognitive science challenge the idea that we can understand the mind by just looking at the brain (Hurley, 1998; Gallagher, 2005; Thompson, 2007; Clark, 2008; Menary, 2010; Shapiro, 2011). They argue that our psychological capacities are realized not just by our brain but also our bodies, as well as the complex ways these bodies interact with their material and social environments. For these “4E” approaches, our psychological capacities are *embodied*, or realized by our physiology and agency; *embedded*, or situated within environmental niches that support and constrain them; *enacted*, or reflective of our first-person activities of sense-making and meaning-creation; and perhaps even *extended* beyond the head by material and social resources around us.

At the heart of 4E approaches is the idea that processes like believing, remembering, and reasoning depend upon resources beyond the head. The artifacts and cultural institutions we use to support these processes are forms of cognitive “scaffolding”: beyond-the-head structures that, when we interact with them, generate ongoing feedback loops that transform our cognitive profile in real-time by opening up new forms of thought and experience.

An important recent development in 4E circles has been an investigation of how moods and emotions might be similarly scaffolded (Griffiths and Scarantino, 2009; Greenwood, 2013; Merritt, 2013; Colombetti, 2014; Krueger, 2014a,b; Slaby, 2014; Stephan et al., 2014; Colombetti and Krueger, 2015; Colombetti and Roberts, 2015; Roberts, 2015; Carter et al., 2016; Krueger and Szanto, 2016). These works examine the many ways we regulate, organize, and maintain our affective life by manipulating everyday artifacts and spaces. We make and listen to music, adorn walls with artworks, consume drugs, wear specific clothing, and gravitate toward spaces and social groups to evoke and regulate different affective experiences. These practices construct “affective niches”: self-styled environments providing the developmental conditions for affective states to take shape and thrive.

“Affectivity Beyond the Skin” is an investigation of some of the many ways affectivity can be scaffolded by resources beyond the head. Each contribution challenges the common view in emotion science that affective states can be characterized by focusing exclusively on internal states of an individual's brain and body.

Wilutzky's rejects the idea that the intentional character of emotions—their being *about* things and states of affairs—is a representational feature of inner mental states. Instead of this headbound picture, Wilutzky develops a rich action-oriented picture of emotional intentionality according to which emotions are actions that guide our dealings with the world and help us learn things about it. As Joona Taipale's tells us, one of the things we learn is how to regulate our affective states in response to the people and things around us. Drawing on an impressive body of developmental literature, Taipale convincingly argues that our ability to self-regulate affect is ontogenetically dependent upon the management and guidance of caregivers.

Paul Elvers and Michelle Maiese continue these explorations in an aesthetic register. Elvers's develops an account of musical self-enhancement. He inaugurates a new research trajectory by showing how music scaffolds feelings of self-esteem and empathy, leading to richer forms of social interaction and interpersonal relatedness. Maiese's is a nuanced investigation of the power of expressive arts like Dance-Movement Therapy to scaffold new ways of inhabiting the world and relating to others. These arts, she argues, enable multifaceted and embodied “affective styles” of moving, speaking, and interacting that can be put to work in the context of therapy and peacebuilding.

Slaby's also explores affective styles—but instead of therapy and peacebuilding, he considers more troubling cultural and political implications. For Slaby, “affective mind invasion” occurs when an individual's affective dispositions are scaffolded by their local subcultures: e.g., corporate workplaces, social web-based groups, academia, the world of sports, or police and military culture. Over time, individuals within these domains habitually—and not always with full awareness and consent—adopt affective styles normative in the domain in question.

Alessandro Salice, Alba Montes Sánchez, Thomas Szanto, and Gavin Sullivan also focus on socially situated affect. Salice and Sánchez's develop a subtle account of “hetero-induced” shame and pride. Against accounts that portray these emotions as essentially self-directed, their account shows how group identification, or the feeling of being interrelated with and evaluated by others, shapes the character and intentional structure of these emotions. Affectivity and group identification is also the topic of Szanto's discussion. Szanto addresses a lacuna in contemporary discussions of akrasia—most of which focus on individual irrationality—with his account of “collaborative” akrasia. For Szanto, core socio-psychological mechanisms of group-level akrasia consist of interpersonally-scaffolded processes of emotion regulation that generate “collaborative spiraling of practical irrationality”: affective feedback loops that reinforce irrational tendencies of group members and animate their behavior. Some similar themes are found in Sullivan's. Drawing upon ethnographic observations of life in downtown Johannesburg before and after the 2010 World Cup in South Africa, Sullivan develops a careful investigation of top-down and bottom-up mechanisms enabling the spread of shared emotions like pride and euphoria.

Finally, Michael's is a brief but substantive discussion of shared emotions. Michael surveys competing accounts of shared emotions, arguing that these accounts are, in fact, not incompatible with one another. Instead of picking out a natural kind, the term “shared emotion” refers to a motley of overlapping phenomena worthy of further interdisciplinary investigation—a fitting coda to this wide-ranging research topic.

It is our hope that this rich and varied collection will serve as the basis for new directions of research investigating affectivity beyond the skin.

## Author contributions

All authors listed have made a substantial, direct and intellectual contribution to the work, and approved it for publication.

### Conflict of interest statement

The authors declare that the research was conducted in the absence of any commercial or financial relationships that could be construed as a potential conflict of interest.
